# The association of maternal anaemia with adverse maternal and foetal outcomes in Somali women: a prospective study

**DOI:** 10.1186/s12905-023-02382-4

**Published:** 2023-04-25

**Authors:** Adil Barut, Deka Omer Mohamud

**Affiliations:** Obstetrics and Gynaecology Department, Somali-Mogadishu Recep Tayyip Erdoğan Research and Training Hospital, Mogadishu, Somalia

**Keywords:** Anaemia incidence, Somalia, Postpartum complications, Moderate anaemia, Severe anaemia

## Abstract

**Background:**

Anaemia in pregnant women is one of the most common public health problems, especially in low- and middle-income countries, such as Somalia. This study aimed to examine the association between the severity of anaemia during pregnancy and the risk of adverse maternal and foetal outcomes in Somali women.

**Methods:**

We prospectively enrolled pregnant women who had deliveries from May 1 to December 1, 2022, at Mogadishu Somali Turkey Recep Tayyip Erdoğan Training and Research Hospital. Blood haemoglobin levels were measured for each participant at admission for delivery. Anaemia was defined as a haemoglobin level of less than 11 g/dL, with mild (10 to 10.9 g/dL), moderate (7 to 9.9 g/dL), and severe (< 7 g/dL) forms. The associations between maternal anaemia and maternal-foetal outcomes were investigated.

**Results:**

The study included 1186 consecutive pregnant women (mean age 26.9 years, range 16–47). The incidence of maternal anaemia at delivery was 64.8%, with 33.8%, 59.8%, and 6.4% of women having mild, moderate and severe forms, respectively. Anaemia at delivery was associated with increased oxytocin administration to prompt labour (OR, 2.25, 95% CI, 1.34–3.78). Both moderate and severe anaemia were associated with increased risks for postpartum haemorrhage (moderate, OR, 4.93; severe, OR, 41.30) and the need for maternal blood transfusions (moderate, OR, 9.66; severe, OR, 301.50). In addition, severe anaemia was associated with increased risks for preterm delivery (OR, 2.50, 95% CI, 1.35–4.63), low birth weight (OR, 3.45, 95% CI, 1.87–6.35), stillbirths (OR, 4.02, 95% CI, 1.79–8.98), placental abruption (OR, 58.04,95% CI, 6.83–493.27) and maternal ICU admission (OR, 8.33, 95% CI, 3.53–19.63).

**Conclusion:**

Our findings suggest that anaemia in pregnancy is associated with adverse maternal and foetal outcomes, with moderate or severe anaemia leading to increased risks for peri-, intra- and postpartum complications and that treatment of severe anaemia in pregnant women should be given particular consideration in our efforts to prevent preterm births, LBW and stillbirths.

## Background

Anaemia is one of the most widespread public health problems, with prevalence rates as high as 30% in women of reproductive age [1]. Anaemia is defined as a condition in which there is less than the normal haemoglobin level, leading to decreased oxygen-carrying capacity of red blood cells in tissues. For pregnant women, the World Health Organization (WHO) defines anaemia as a haemoglobin concentration of less than 11 g per decilitre (g/dL), with severity levels classified as mild, moderate, and severe [2].

Anaemia is among the most common conditions that affect pregnancies, with varying incidences and aetiologies, depending on the geographic location [1, 2]. The incidence of anaemia throughout pregnancy has been reported to be 32 to 52% in developing countries compared with only 15 to 23% in developed countries. The WHO estimates that over 50% of pregnant women in underdeveloped countries are anaemic. West-central Africa and South Asia are particularly notorious for anaemia cases and resultant complications [1].

The causes of anaemia in pregnancy include unhealthy lifestyle, pregnancy itself, alcohol intake, smoking, malnutrition, blood loss, chronic diseases, and chronic infections. Other risk factors contributing to the high burden of anaemia among pregnant women in developing countries include low socioeconomic status, rural residence, short birth intervals, late initiation of antenatal medications, grand multiparity, the third gestational trimester, and deficiencies of vitamin B_12_, vitamin A, riboflavin, folic acid, and iron. In Sub-Saharan Africa, dietary iron deficiency is the most common cause of anaemia in pregnancy, justifying the term iron deficiency anaemia [3, 4].

Anaemia during pregnancy, particularly severe anaemia, is not only a major cause of morbidity and mortality in pregnant women in low- and middle-income countries (LMIC), but also has been associated with increased risks of both maternal and neonatal adverse outcomes. It is also an important predictor of poor pregnancy outcomes, such as low birth weight (LBW), prematurity, stillbirths, intrauterine growth restriction, abortions, antepartum haemorrhage, postpartum haemorrhage, preeclampsia, and prolonged labour [5].

There have been few data on the prevalence of maternal anaemia and on its association with maternal and foetal outcomes among Somali women. A meta-analysis from Ethiopia of 20 studies found a pooled prevalence of anemia among pregnant women as 31.66%, with the highest prevalence of 56.80% in Ethiopia Somali region [6].

Furthermore, because of the large sample size requirements, little is known regarding the association between maternal anaemia and adverse outcomes. This insufficiency of pertinent data provided the rationale forthis study to examine the association between the severity of anaemia during pregnancy and the risk of adverse maternal and foetal outcomes in Somalia.

## Materials and methods

### Study design and participants

This prospective study included data on 1283 consecutive mothers who had deliveries from May 1, 2022, to December 1, 2022, at the department of obstetrics of Mogadishu Somali Turkey Recep Tayyip Erdoğan Training and Research Hospital in Mogadishu, the capital city of Somalia. As a tertiary care facility performing nearly 3,000 deliveries annually, it is a dedicated centre for high-risk and referred cases.

Data on pregnant women were prospectively recorded from admission to discharge, including maternal age, parity, gestational age, APGAR score, maternal blood haemoglobin level at delivery, birth weight, delivery method, stillbirths, new-borns requiring neonatal intensive care unit (NICU), preterm delivery, oxytocin administration for augmentation of labour, foetal gross congenital anomalies, foetal distress, emergency primary caesarean section, prolonged labour, severe preeclampsia or eclampsia, antepartum haemorrhage (placenta previa, placental abruption), maternal blood transfusions (need for blood transfusion during/within 24 h of delivery), in-hospital maternal mortality, maternal admission intensive care unit (ICU), and postpartum haemorrhage. All women underwent ultrasound examination at admission. Gestational age was calculated from the last menstrual period in combination with obstetric ultrasound examination done before 20 weeks of pregnancy. In case of no early ultrasound and unknown last menstrual period, gestational age was estimated based on ultrasound examination. Before initiation of enrolment and data recording, the residents and midwives were trained by the first author (A.B) about how to meet the pregnant women, what to ask them, what information to be elicited and how to lead the patients throughout the delivery process. In addition, appropriate data recording on Excel data sheets was delineated.

Inclusion criteria were age 16–47 years, singleton pregnancy, and documented placental diseases (preeclampsia, placenta previa, placental abruption) or foetal gross congenital anomalies shortly before or at the time of delivery. Women were excluded in the presence of any of the following: multi gestation, incomplete clinical or hospital data, chronic hypertension, malaria, and diabetes mellitus. Twenty-two women who did not give consent to be enrolled in the study were also excluded. Data from 46 women could not be completed and were considered missing. These patients were not included in the final analysis.

The study was approved by the Mogadishu Somali Turkey Recep Tayyip Erdoğan Training and Research Hospital Ethics and Research Committee (Permission number: MSTH/10205/18.04.2022/547). The study was performed in accordance with the principles and guidelines of the Declaration of Helsinki. All participants were informed about the study and gave consent to publication of the results. Analysis and reporting of the results are in compliance with the Strengthening the Reporting of Observational Studies in Epidemiology (STROBE) checklist.

### Definitions

Haemoglobin levels are routinely obtained at admission of pregnant women. Maternal anaemia and LBW were defined according to the WHO criteria; that is a blood haemoglobin level of less than 11 g/dL, with mild (Hb 10 to 10.9 g/dL), moderate (Hb 7 to 9.9 g/dL), and severe (Hb < 7 g/dL) forms, and a birth weight of less than 2,500 g for LBW. Stillbirth was defined as death before or during delivery after 20 weeks of pregnancy.

### Data processing and analysis

For data collection, a structured format was used including all relevant clinical information. Data were processed using the Statistical Package for Social Sciences (SPSS) version 21 (IBM Corp., Armonk, N.Y.; USA). Quantitative data were expressed as means, standard deviation (SD), median, minimum, and maximum, and qualitative data as frequencies and percentages. Homogeneity was checked using Levene's test, with a *p* value of > 0.05 considered in favour of homogeneity. The Shapiro–Wilk normality test was used to check whether continuous variables were normally distributed.

For pairwise comparisons, numerical variables were compared using the independent t-test if normally distributed. Multigroup comparisons of normally distributed variables were made using the one-way ANOVA test. The Post Hoc Multiple Comparisons (Bonferroni) were used to determine between-group differences. Nominal variables were analysed with Pearson's or Fisher's chi-squared test. Multinominal univariate logistic regression analysis was performed to determine the risk for maternal and foetal parameters in relation to laboratory parameters. A *p* value of less than 0.05 was accepted as statistically significant. All variables were expressed with 95% confidence intervals (CI).

## Results

### Sociodemographic and obstetric characteristics

During the study period, 1283 women were admitted for delivery, of whom 1186 were eligible for the analysis. A total of 97 women were excluded because of multi gestation (*n* = 8), chronic hypertension (*n* = 18), malaria (*n* = 11), diabetes mellitus (*n* = 38) and women who did not give consent (*n* = 22). The characteristics of the participants are summarised in Tables 1 and 2. The mean age was 26.9 ± 5.7 years (range 16–47). The median parity was 3 (range 1–14). Delivery occurred at a mean of 37.4 ± 3.47 weeks. The mean birth weight was 2,952 ± 714 g. A total of 123 women had no early ultrasound examination or could not remember their last menstrual period; therefore, gestational age was estimated based on ultrasound examination.

**Table 1:** Clinical characteristics

**Variables**	Mean ± SD	number	percent)
Age	26.9 ± 5.7		
At delivery week	37.4 ± 3.5		
Overall haemoglobin level g/ dL	10.2 ± 1.7		
Non-anaemia	12.0 ± 0.8	417	35.2
Anaemia, g/dL	9.2 ± 1.3	769	64.8
Mild	10.5 ± 0.3	260	33.8
Moderate	8.9 ± 0.7	460	59.8
Severe	6.0 ± 1.0	49	6.4
Overall birth weight	2914 ± 774		
LBW	1842 ± 599	280	23.6
APGAR score			
1.minute	7.6 ± 1.1		
5.minute	8.5 ± 1.1		

*SD* standard deviation min: minimum, max: maximum, *LBW* Low birth weight

**Table 2:** Maternal and foetal outcomes

Delivery method	Number	Percent
Normal vaginal delivery	725	61.1
C-section	461	38.9
**Complications**		
Stillbirth	89	7.5
NICU admission	150	12.6
Preterm delivery	275	23.2
Oxytocin administration to prompt labours	96	13.1
Foetal gross congenital anomalies	11	0.9
Foetal distress	62	5.2
Emergency primary C-section	196	16.5
Prolonged labour	36	3.87
Severe preeclampsia or eclampsia	85	7.2
Placenta previa	7	0.6
Placental abruption	13	1.1
Maternal blood transfusion	57	4.8
In-hospital maternal mortality	8	0.75
Maternal admission ICU	60	5.1
Postpartum haemorrhage	41	3.5

*NICU* Neonatal intensive care unit

### Incidence of anaemia

**Fig. 1:**
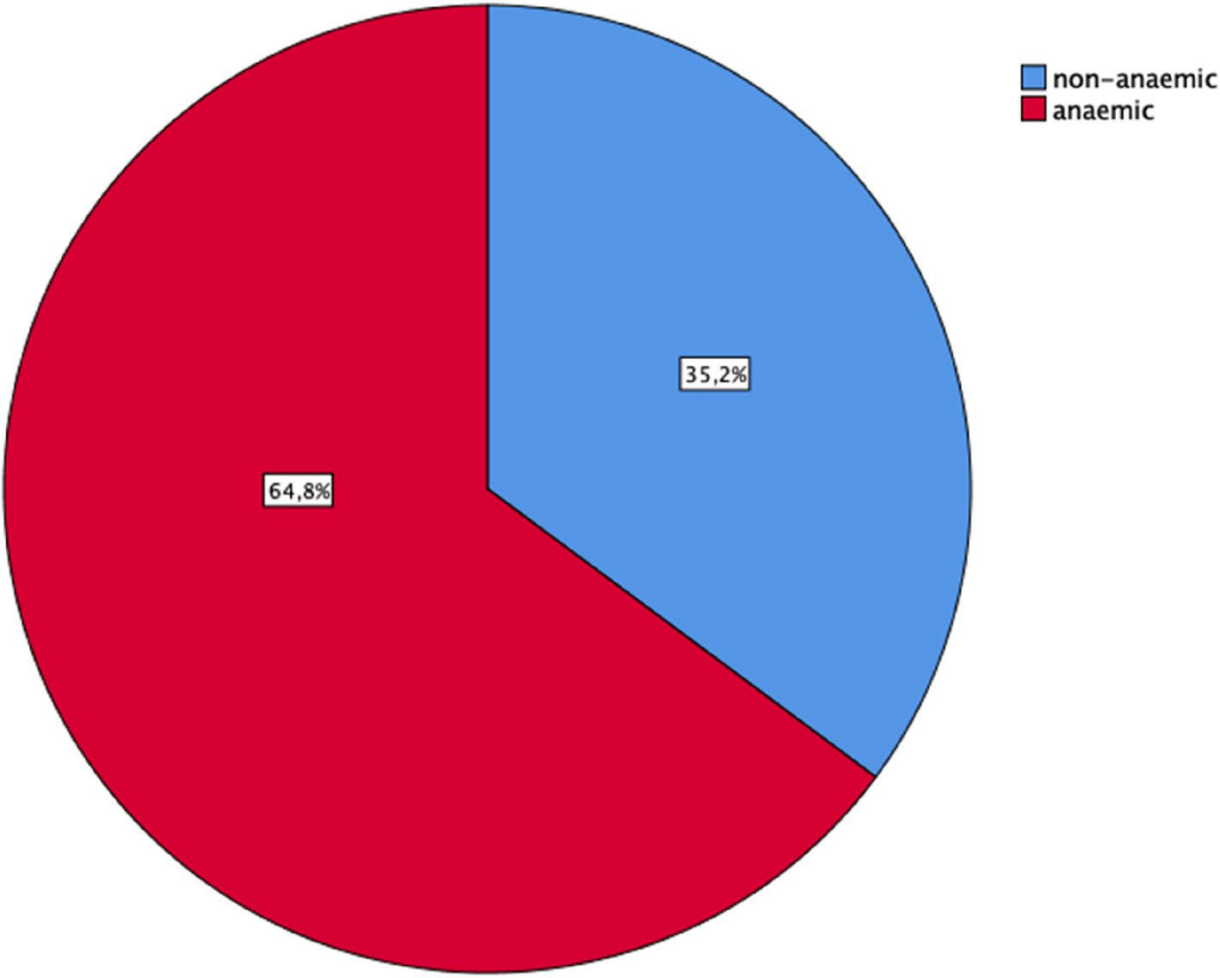
Pie chart showing the overall prevalence of anaemia

The mean haemoglobin level was 10.2 ± 1.7 g/ dL (range 2.7– 14.5 g/dL: reference range 11 g/dL). Of 1186 women, 769 (64.8%) had Anaemia of varying severity (Fig. 1). The majority of anaemic patients (59.8%, 460/769) had moderate anaemia, followed by mild (33.8%, 260/769) and severe (6.4%, 49/769) forms (Table 1).

### Between-group comparisons

A comparison between anaemic and non-anaemic mothers showed no significant between-group differences with respect to maternal factors (age, preterm delivery, severe preeclampsia or eclampsia, in-hospital maternal mortality, emergency caesarean section, prolonged labour) and foetal characteristics (birth weight, LBW, APGAR score, stillbirths, foetal NICU, foetal gross congenital anomalies, and foetal distress). Mothers with anaemia significantly differed from those without anaemia with respect to an increased incidence of placental abruption (1.6% vs 0.2%, *p* = 0.037), postpartum haemorrhage (4.8% vs 1% *p* = 0.001), maternal blood transfusions (7.4% vs 3.6% *p* = 0.009), maternal ICU admission (6% vs 3.4% *p* = 0.049), and oxytocin administration to prompt labour (16% vs 7.8% *p* = 0.002), but significantly decreased delivery weeks (37.3 vs 37.7 *p* = 0.049) (Table 3).

**Table 3:** Comparison of anaemic and non-anaemic women with respect to maternal and foetal clinical characteristics

**Parameters**	**Anaemic *n* = 769** mean ± SD	**Non-anaemic *n* = 417** mean ± SD	**T**	***P* value**
Maternal Age (years)	26.8 ± 5.8	27.1 ± 5.7	0.987^a^	0.324
Delivery weeks	37.3 ± 3.6	37.7 ± 3.1	2.010^a^	0.045*
Birth weight	2882 ± 818	2970 ± 694	1.863^a^	0.063
Apgar (1. minute)	7.6 ± 1.1	7.6 ± 1.1	0.456^a^	0.649
Apgar (5. minute)	8.5 ± 1.1	8.6 ± 1.1	0.712^a^	0.477
**Fetal outcomes**	**n (%)**	**n (%)**	**X**^**2**^	***p***
LBW	195(25.4)	85(20.4)	3.709^b^	0.054
Preterm delivery	185(24.1)	90(21.6)	0.930 ^**b**^	0.335
Stillbirth	64(8.3)	25(6.0)	2.110 ^**b**^	0.146
Foetal NICU admission	107(15.2)	43(11)	3.78^b^	0.052
Foetal gross congenital anomalies	8(1)	4(1)	0.007 ^b^	0.933
foetal distress	35(4.6)	27(6.5)	2.019 ^b^	0.155
**Matenal outcomes**				
Severe preeclampsia or eclampsia	53(6.9)	32(7.7)	0.248 ^**b**^	0.618
Placental abruption	12(1.6)	1(0.2)	4.350^b^	0.037*
Postpartum haemorrhage	37(4.8)	4(1)	12.022^b^	0.001*
Maternal blood transfusion	57(7.4)	15(3.6)	6.901 ^b^	0.009*
Maternal ICU admission	46(6.0)	14(3.4)	3.877 ^b^	0.049*
In-hospital maternal mortality	5(0.7)	3(0.7)	0.019 ^b^	0.889
foetal distress	35(4.6)	27(6.5)	2.019 ^b^	0.155
Emergency caesarean section	129(21.5)	67(20.7)	0.065 ^**b**^	0.798
Prolonged labour	25(4.2)	11(3.4)	0.370^b^	0.543
Oxytocin administration to prompt labour	76(16)	20(7.8)	9.822 ^**b**^	0.002*

*LBW* Low birth weight,

^b^ chi-square test

^a^ Independent-Samples T-Test

^*^ *p* < 0.01, SD: standard deviation

In addition, non-anaemic mothers did not differ significantly from mothers with varying forms of anaemia (mild, moderate, and severe) with respect to maternal factors (maternal age, severe preeclampsia or eclampsia, in-hospital maternal mortality, emergency caesarean section, and prolonged labour) and foetal characteristics (APGAR score, NICU admission, foetal gross congenital anomalies, and foetal distress). However, as compared with mothers with severe anaemia, non-anaemic mothers had significantly lower rates of LBW (20.4% vs 46.9%, *p* = 0.0001), preterm delivery (21.6% vs 40.8%, *p* = 0.0006), placental abruption (0.2% vs 12.2%, *p* = 0.0001), maternal blood transfusions (3.6% vs 93.9%, *p* = 0.0001), maternal admission ICU care (3.4% vs 22.4%, *p* = 0.0001), stillbirths (6% vs 20.4%, *p* = 0.0001), but significantly increased delivery weeks (37.7 vs 35.9, *p* = 0.004) and birth weight (2,969 g vs 2,598 g, *p* = 0.009). In addition, mothers with severe or moderate anaemia significantly differed from those without anaemia with respect to an increased incidence of postpartum haemorrhage (28.6% and 4.6% vs 1%, respectively; *p* = 0.0001, *p* = 0.001), and oxytocin administration to prompt labour (24.1% and 15.5% vs 7.8%, respectively; *p* = 0.009) (Table 4).

**Table 4:** Comparisons across non-anaemic and anaemic groups

Parameters	No-anaemia	Mild	Moderate	Severe	F	P
	Mean ± SD	Mean ± SD	Mean ± SD	Mean ± SD		
Maternal age	27.1 ± 5.6	27.0 ± 5.3	26.7 ± 6.0	27.3 ± 6.8	0.518^a^	0.765
Delivery (Gestational week)	37.7 ± 3.13	37.7 ± 3.8	37.2 ± 3.4	35.9 ± 4.5	5.277^a^	P1:1 P2:0.170 P3:0,004*
Birth weight g	2969 ± 691	2941 ± 796	2881 ± 814	2598 ± 873	3.832^a^	P1:1 P2:0.554 P3:0,009*
Apgar (1. minute)	7.6 ± 1.1	7.6 ± 1.1	7.6 ± 1.1	7.6 ± 1.0	0.084^a^	0.969
Apgar (5. minute)	8.6 ± 1.1	8.5 ± 1.1	8.5 ± 1.1	8.6 ± 1.1	0.273^a^	0.845
**Foetal outcomes**	**n (%)**	**n (%)**	**n (%)**	**n (%)**	**X**^**2**^	
LBW	85(20.4)	55(21.2)	117(25.4)	23(46.9)	18.912^b^	P1 > 0.05 P2 > 0.05 P3:0.0001**
Preterm delivery	90(21.6)	50(19.2)	115(25)	20(40.8)	12.287^b^	P1 > 0.05 P2 > 0.05 P3:0.006*
Stillbirth	25(6	12(4.6)	42(9.1)	10(20.4)	18.001^b^	P1 > 0.05 P2 > 0.05 P3:0.0001**
NICU admission	43(11)	39(15.7)	61(14.6)	7(17.9)	4.22^b^	0.239
Foetal gross congenital anomalies	4(1,00)	4(1.5)	3(0.7)	0(0,00)	1.899^b^	0.594
Foetal distress	27(6.5)	17(6.6)	16(3.5)	2(4.1)	5.182^b^	0.159
**Maternal outcomes**						
Severe preeclampsia or eclampsia	32(7.7)	14(5.4)	34(7.4)	5(10.2)	2.117^b^	0.549
Placental abruption n	1(0.2)	2(0.8)	4(0.9)	6(12.2)	66.241^b^	P1 > 0.05 P2 > 0.05 P3:0.0001**
Postpartum haemorrhage	4(1)	2(0.8)	21(4.6)	14(28.6)	107.718^b^	P1 > 0.05 P2:0.001 P3:0.0001**
Maternal blood transfusion	15(3.6)	2(0.8)	9(2)	46(93.9)	693.472^b^	P1 > 0.05 P2 > 0.05 P3:0.0001**
Maternal ICU admission	14(3.4)	7(2.7)	28(6.1)	11(22.4)	37.409b	P1 > 0.05 P2 > 0.05 P3:0.0001**
In-hospital maternal mortality	3(0.7)	1(0.4)	3(0.7)	1(0.2)	0.933^b^	0.818
Emergency caesarean section	67(20.7)	47(23.5)	71(19.6)	11(28.2)	2.364^b^	0.50
Prolonged labour	11(3.4)	11(5.5)	12(3.3)	2(5.1)	2.126^b^	0.547
Oxytocin administration to prompt labours (%)	20(7.8)	24(15.4)	45(15.5)	7(24.1)	11.634^b^	P1 > 0.05 p2:0.009* P3: 0.009*

P1:no-anaemia-mild, p2:no-anaemia-moderate, p3:no-anaemia-severe

^a^ chi-square test

^b^ One-Way ANOVA test (Bonferroni), b: chi-square test SD: standard deviation

^*^ *p* < 0.01

^**^p < .001

### Univariate analysis

Results of the univariate analysis as summarised in Table 5. Maternal anaemia, moderate anaemia, and severe anaemia were significantly associated with postpartum haemorrhage and maternal blood transfusions. Both maternal anaemia and all forms of anaemia were significantly associated with oxytocin administration to prompt labour. Severe anaemia was significantly associated with preterm delivery, LBW, stillbirths, placental abruption, and maternal ICU admission.

**Table 5:** Associations of anaemia and anaemia severity forms with maternal and foetal adverse outcomes

	**OR (CI 95%)**			
**Maternal outcomes**	**Anaemia vs no-anaemia**	**Mild vs no-anaemia**	**Moderate vs no-anaemia**	**Severe vs no-anaemia**
Preterm delivery	1.15(0.86–1.53) *p*:0.335	1.15 (0.78–1.70) *p*:0.865	1.21(0.88–1.66) *p*: 0.233	2.50(1.35–4.63) *p*:0.003*
Severe preeclampsia or eclampsia	1.12(0.71–1.77) *p*: 0.618	1.46(0.76–2.79) *p*: 0.252	1.04(0.63–1.72) *p*: 0.874	1.36(0.50–3.69) *p*: 0.537
Placental abruption	6.59(0.85–50.87) *p*: 0.070	3.22(0.29–35.73) *p*: 0.340	3.64(0.40–32.76) *p*: 0.248	58.04(6.83–493.27) *p*:0.0001**
Postpartum haemorrhage	5.21(1.84–14.74) *p*: 0.002*	1.24(0.22–6.86) *p*: 0.798	4.93(1.68–14.50) *p*: 0.004*	41.3(12.90–132.20) *p*: 0.0001**
Maternal blood transfusions^a^	2.14(1.19–3.83) *p*: 0.010*	1.52(0.69–3.35) *p*: 0.296	9.66(1.26–73.60) p: 0.029*	301.5(95–947) p: 0.0001**
Maternal ICU admission	1.83(0.99–3.37) *p*: 0.052	1.25(0.50–3.15) *p*: 0.628	1.86(0.96–3.59) *p*: 0.062	8.33(3.53–19.63) *p*: 0.0001**
In-hospital maternal mortality	1.11(0.26–4.66) *p*: 0.889	1.87(0.19–18.14) *p*: 0.544	1.1(0.22–5.50) *p*: 0.904	2.87(0.29–28.18) *p*: 0.365
Emergency caesarean section	1.03(0.73–1.46) *p*: 0.848	1.21(0.79–1.88) *p*: 0.371	1.19(0.80–1.78) *p*: 0.370	1.06(0.41–2.71) *p*: 0.898
Prolonged labour	1.25(0.60–2.57) *p*: 0.544	1.67(0.71–3.94) *p*: 0.236	1.01(0.44–2.33) *p*: 0.977	1.55(0.33–7.30) *p*: 0.574
Oxytocin administration to prompt labour	2.25(1.34–3.78) *p*: 0.002*	2.15(1.14–4.04) *p*: 0.017*	2.16(1.24–3.78) *p*: 0.006*	3.77(1.43–9.89) *p*: 0.007*
**foetal outcomes**				
LBW	1.32(0.99–1.77) *p*: 0.055	1.04 (0.71–1.53) *p*: 0.810	1.33 (0.97–1.83) *p*: 0.077	3.45 (1.87–6.35) *p*: 0.0001**
Stillbirth	1.42(0.88–2.29) *p*:0.148	1.31(0.65–2.67) *p*:0.444	1.57(0.94–2.63) *p*:0.083	4.02(1.79–8.98) *p*:0.0001**
NICU admission	1.45(0.99–2.11) *p*: 0.053	1.51(0.95–2.41) *p*: 0.081	1.38(0.91–2.10) *p*: 0.124	1.77(0.73–4.26) *p*: 0.20
Foetal gross congenital anomalies	1.08(0.32–3.62) *p*: 0.894	1.61(0.39–6.50) *p*: 0.501	1.47(0.32–6.63) *p*: 0.612	2.15(0.23–19.63) *p*: 0.497
Foetal distress	1.45(0.86–2.43) *p*: 0.157	1.01(0.53–1.89) *p*: 0.974	1.92(1.02–3.62) *p*: 0.430	1.62(0.37–7.06) *p*: 0.516

*OR* Odds Ratio, reference: no-anaemia, *NICU* Neonatal intensive care unit, *LBW* Low birth weight (< 2,500 g),

^a^Maternal blood transfusion: Need for blood transfusion during/within 24 h of delivery

^*^p < 0,05

^**^p < 0,01

## Discussion

In our study, we evaluated the associations between maternal anaemia at delivery and in-hospital maternal and foetal outcomes in Somali women. To our knowledge, this is the largest series of pregnant women to examine the impact of maternal anaemia at delivery on adverse maternal and foetal outcomes in the Sub-Saharan Africa region. The prevalence of anaemia at delivery was alarmingly high at 64.8%, representing the second highest rate across all African countries, after that reported from the Republic of Benin (68.2%) [7], a relatively small west African country with a smaller population as compared with Somalia (≈13 vs 17 million). In Somalia, the prevalence of anaemia among pregnant women was reported to be 49% by the World Bank report 2019 (https://data.worldbank.org/indicator/SH.PRG.ANEM?locations=SO). A recent systematic review and meta-analysis for Sub-Sharan Africa countries reported varying rates of anaemia among pregnant women with considerable provincial differences, i.e., Ethiopia (7.9- 53.9%), Nigeria (46.6–76.9%), Ghana (41.5–76%), Kenya (36.2–57%), and Uganda (22.1–32.5%) [7]. A meta-analysis of 20 studies from Ethiopia reported the prevalence of anemia as 31.66% in pregnant women, with the highest prevalence of 56.80% in Ethiopia Somali region [6].

In this study, the majority of anaemic patients (59.8%) had moderate anaemia, followed by mild (33.8%) and severe (6.4%) forms (Table 1), as compared with 30.6%, 25.3% and 1.2%, respectively, reported for Tanzania [8] and 39%, 21.1%, and 12%, respectively, reported for Egypt [9].

In relation to maternal outcomes, our study demonstrated significant differences between anaemic and non-anaemic women with respect to the development of postpartum haemorrhage and placental abruption as well as the need for maternal blood transfusions, ICU admission, and oxytocin administration to prompt labour. In univariate analysis, maternal anaemia was found to be significantly associated with postpartum haemorrhage, maternal blood transfusions, and oxytocin administration.

Moreover, in univariate analysis, moderate, not mild, maternal anaemia was found to be significantly associated with increased risks of postpartum haemorrhage (OR 4.93), maternal blood transfusions (OR 9.66), oxytocin administration (OR 2.15). More importantly, with severe maternal anaemia, the number of ensuing risks considerably increased, including preterm delivery (OR 2.50), LBW (OR 3.45), stillbirth (OR 4.02), placental abruption (OR 58.04), and postpartum haemorrhage (OR 41.30), as well as the need for maternal blood transfusions (OR 301.50), maternal ICU admission (OR 8.33), and oxytocin administration (OR 3.77).

A systematic review and meta-analysis that included 26 studies of anaemic pregnant women in LMICs found significantly higher risk ratios (RR) for low birth weight (RR: 1.3), preterm birth (RR: 1.63), and perinatal mortality (RR: 1.51) [10]. In another systematic review and meta-analysis that included 148 studies, maternal anaemia was reported to be predictive of postpartum haemorrhage, blood transfusions, preeclampsia, LBW, preterm birth, stillbirth, and perinatal mortality [11]. In a study from China, the presence of anaemia (mild, moderate, or severe) during pregnancy was associated with increased risks for placental abruption and severe postpartum haemorrhage and moderate or severe anaemia with increased risks for stillbirth, preterm birth, and maternal death [12]. In another study from Gambia, severe maternal anaemia was associated with stillbirths, LBW, NICU admissions, and preterm deliveries [13]. Similarly, pregnancies of women with severe anaemia from Pakistan and India were complicated by stillbirth, preterm delivery, LBW, and postpartum haemorrhage [14]. Adverse effects of maternal anaemia also included pregnancy-induced hypertension in Nepali women, along with postpartum haemorrhage and ICU admission [15].

The current study clearly shows that determination of the severity levels of anaemia among pregnant women particularly during the last trimester is important in LMICs, particularly in poverty-stricken African countries with limited sources to counteract pregnancy-related health issues. Thus, given the severity of anaemia, preventive and therapeutic interventions are required, including dietary habits and iron supplementation, for which international solidarity and aid are of particular importance.

### Limitations

Although our study provides clear-cut data about the current condition of pregnancies among Somali women and adverse consequences of maternal anaemia and despite its considerably large sample size, it reflects single-centre experience and thus may not be representative of the general population.

## Conclusion

Our findings suggest that anaemia in pregnancy is associated with adverse maternal and foetal outcomes, with moderate or severe anaemia leading to increased risks for peri-, intra- and postpartum complications and that treatment of severe anaemia in pregnant women should be given particular consideration in our efforts to prevent preterm births, LBW and stillbirths.

## Data Availability

All data generated or analyzed during this study are included in this article. The datasets used and/or analyzed during the current study available from the corresponding author on reasonable request, but restrictions apply to the availability of these data, which were used under license for the current study, and so are not publicly available. Corresponding author (email: dradilbarut@gmail.com) can be contacted for the data with a reasonable request.

## Abbreviations

OR: Odds ratio
CI: Confidence interval
Hgb: Haemoglobin
OR: Odds ratio
SPSS: Statistical package for social science
WHO: World Health Organization
LMIC: Low- and middle-income countries
LBW: Low birth weight
NICU: Neonatal intensive care unit
ICU: Intensive care unit
SD: Standard deviation
